# AAA Proteases: Guardians of Mitochondrial Function and Homeostasis

**DOI:** 10.3390/cells7100163

**Published:** 2018-10-11

**Authors:** Magdalena Opalińska, Hanna Jańska

**Affiliations:** Department of Cellular Molecular Biology, Faculty of Biotechnology, University of Wroclaw, F. Joliot-Curie 14A, 50-383 Wroclaw, Poland

**Keywords:** AAA protease, ATP-dependent proteolysis, mitochondria, inner mitochondrial membrane proteostasis, *m*-AAA protease, *i*-AAA protease, neurodegenerative diseases

## Abstract

Mitochondria are dynamic, semi-autonomous organelles that execute numerous life-sustaining tasks in eukaryotic cells. Functioning of mitochondria depends on the adequate action of versatile proteinaceous machineries. Fine-tuning of mitochondrial activity in response to cellular needs involves continuous remodeling of organellar proteome. This process not only includes modulation of various biogenetic pathways, but also the removal of superfluous proteins by adenosine triphosphate (ATP)-driven proteolytic machineries. Accordingly, all mitochondrial sub-compartments are under persistent surveillance of ATP-dependent proteases. Particularly important are highly conserved two inner mitochondrial membrane-bound metalloproteases known as *m*-AAA and *i*-AAA (ATPases associated with diverse cellular activities), whose mis-functioning may lead to impaired organellar function and consequently to development of severe diseases. Herein, we discuss the current knowledge of yeast, mammalian, and plant AAA proteases and their implications in mitochondrial function and homeostasis maintenance.

## 1. Introduction

Mitochondria are multifunctional organelles that play a central role in a broad range of life-sustaining tasks within eukaryotic cells, including adenosine triphosphate (ATP) production, calcium storage, and cofactor-generating pathways such as iron-sulfur cluster biogenesis [1,2,3,4]. Mitochondria are also key mediators in cell proliferation, differentiation, and death signaling as well as in innate immunity [2,3,5,6]. Consequently, disturbances in mitochondrial homeostasis and activity lead to cellular pathologies that are linked with the onset of many severe diseases [1,7,8]. 

Broad arrays of mitochondrial functions are accomplished by multifaceted proteome comprised of about 1500 polypeptides that are spread between aqueous and membranous mitochondrial sub-compartments: Outer membrane (OM), intermembrane space (IMS), inner membrane (IM), and matrix [8,9]. Optimal functioning of mitochondria largely depends on accurate composition of mitochondrial proteome and its quality [10,11,12,13]. Consequently, mitochondrial protein content undergoes persistent adaptations to meet cellular needs and quality control. From this perspective, IM is particularly challenging mitochondrial sub-compartment. Up to 40% of the total mitochondrial proteome is accommodated in IM, including electron transport chain and F_1_-F_0_ ATP synthase (oxidative phosphorylation (OXPHOS) system). This renders IM one of the most protein-rich membranes known [14]. In addition to the extremely crowded milieu, the bi-genomic nature of mitochondrial proteome challenges the biogenesis of many IM-bound protein complexes [15,16,17]. While the majority of the mitochondrial proteome is encoded by nuclear genome, synthesized in the cytosol and subsequently imported into the organelle, and a limited number of mitochondrial proteins (~1%, including few subunits of OXPHOS complexes) are encoded by mitochondrial genome (mtDNA) in the organellar matrix [15,16,17]. This imposes a need for precisely synchronized expression, sorting and folding of both nuclear and mitochondrial encoded subunits to enable their subsequent assembly into functional stoichiometric complex in IM. Additional challenges in the biogenesis of the vital IM-bound complexes include insertion of indispensable redox cofactors into their proteinaceous structure. The failure in this process abolishes further maturation of the complex and is linked to the generation of harmful reactive oxygen species (ROS) [18,19,20]. While mitochondria are equipped with numerous free radical-scavenging mechanisms, mitochondrial proteome is continuously exposed to the deleterious ROS molecules that arise as byproducts of OXPHOS activity [21]. ROS can directly damage mitochondrial proteins or introduce mutations in mitochondrial genome rising the risk of perturbations in the folding of mtDNA-encoded subunits [22]. 

The particular milieu of IM requires constant monitoring for damaged, unassembled, and superfluous proteins. The pivotal role in this process play two IM-bound ATP-dependent proteases that belong to the FtsH (Filamentous temperature sensitive H) family of peptidases: *m*-AAA (matrix) and *i*-AAA (intermembrane space) proteases (known as AAA proteases). Remarkably, the role of these ATP-fueled proteolytic machines is not limited to IM protein quality control [10,23,24,25]. This review summarizes the current state of knowledge on pleiotropic functions of AAA proteases and their implications for mitochondrial functions and homeostasis.

## 2. Molecular Architecture and Mode of Action of AAA Proteases

**Figure 1:**
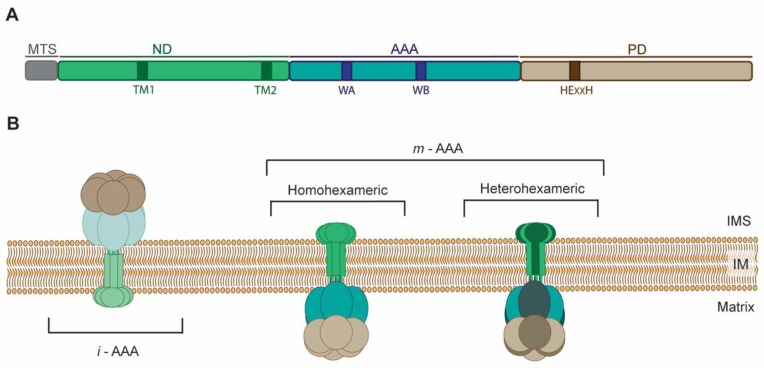
Structural characteristics and organization of AAA (ATPases associated with diverse cellular activities) proteases in mitochondrial inner membrane. (**A**) Domain architecture of AAA proteases. Subunit of *m*-AAA protease is presented on the scheme. In contrast to *m*-AAA, subunit of *i*-AAA protease in the N-terminal domain (ND) contains only one transmembrane region (TM). MTS, mitochondrial targeting sequence; AAA, AAA domain; PD, proteolytic domain; WA and WB, Walker A and Walker B motifs. (**B**) Topology of oligomers of AAA proteases in the inner mitochondrial membrane. IMS, intermembrane space; IM, inner membrane.

Mitochondrial AAA proteases belong to AAA+ (ATPases associated with diverse cellular activities) superfamily that couple energy derived from ATP hydrolysis to versatile functions. The hallmark of the members of the AAA+ superfamily is the characteristic P-looped AAA domain that is required for the ATPase activity [26]. Mitochondrial AAA proteases are counterparts of the prokaryotic membrane-bound FtsH. All these proteins form membrane-embedded complexes, where the single subunit can be divided into: N-terminal domain (ND) with insoluble membrane-bound region(s) (TM); P-looped AAA domain (AAA) containing the nucleotide-binding Walker A and B motifs responsible for ATP binding and hydrolysis, respectively; proteolytic chamber-forming M41 metallopeptidase domain with a conserved Zn^2+^ binding HExxH (His-Glu-x-x-His) motif serving as an active site (PD) [27] (Figure 1A). Six multidomain subunits assemble and give rise to functional *m*-AAA or *i*-AAA proteases. Oligomers are composed of stacked AAA and protease hexameric rings forming double donut-like structures that are anchored in IM by TM [28,29] (Figure 1B). Mitochondria of all eukaryotes contain homomeric *i*-AAA protease (Figure 1B), which is formed by oligomers of YME1L in mammals and Yme1 in yeast [30]. Interestingly, plant mitochondria contain up to two *i*-AAA proteases composed of either FTSH4 or FTSH11 subunits, of which the FTSH11 complex is also found in chloroplasts [31]. On the contrary, subunits of *m*-AAA occur as multiple isoforms that arrange, depending on species and tissue, into homo or heteromeric complexes (Figure 1B). For instance, in yeast Yta10 and Yta12 assemble together into functional *m*-AAA protease [32]. In humans, the protease comprises either from AFG3L2 subunits only, or heterohexamers of AFG3L2 and paraplegin (known also as SPG7) subunits [33]. Mice express another isoform of *m*-AAA subunit AFG3L1, which, like AFG3L2, is able to form homo or heterohexamers with AFG3L2 and paraplegin expanding the repertoire of these ATP-fueled proteolytic machines even further [33,34]. Interestingly, *Afg3l1* encoding AFG3L1 is a pseudogene is humans [34]. Plant *m*-AAA complexes are composed of FTSH3 and FTSH10 subunits that can either form homo or heterooligomers [35]. Association between AAA proteases subunits is mainly driven by interactions between their ATPase domains [36,37]. However, interactions within other domains are also important for the oligomerization. For instance, lack of ND containing TM abolishes assembly of human *i*-AAA subunits, while substitution of TM with the artificial hexamerization motif (*cc-hex* sequence) permits subunits oligomerization and proteolytic activity [37]. In contrast, yeast *m*-AAA subunits, devoid of TM regions, assemble into functional proteolytic complex [28]. In this case, the interactions between metallopeptidase domains are stabilizing *m*-AAA oligomers.

The recent elegant study of the yeast *i*-AAA protease structure showed that ATPase domains create irregular spiral staircase on the top of planar protease ring. Conserved tyrosine residues in P-loop of ATPase domain that are exposed into central pore mediate ATP hydrolysis cycle-dependent stepwise substrate translocation into negatively charged proteolytic chamber [29]. The maximized unfolding force with concomitant maintenance of the grip of the translocating substrate is ensured by the coordinated ATP hydrolysis, where the binding of ATP inhibits ATP hydrolysis in neighboring subunit resulting in sequential hydrolysis cycle in AAA ring [38]. Interestingly, the degree of ATP hydrolysis coordination between adjacent subunits diverges in AAA proteases from different species [23,38]. In the case of human *m*-AAA protease homo and heterooligomers, binding of ATP to either AFG3L2 or paraplegin inhibits ATP hydrolysis in neighboring subunits. This is different in yeast, where binding of ATP only to Yta12 subunit blocks ATP hydrolysis in adjacent Yta10, not the other way around [38].

To limit the risk of harmful uncontrolled protein degradation, recognition of substrates by AAA proteases needs to be precisely controlled. AAA proteases seem to recognize the folding state of a solvent-exposed domain of the substrate [36]. The minimal length of the sequence that needs to protrude from the membrane in order to enable substrate recognition is ranging from 10 to 20 amino acids [39]. AAA proteases display a degenerate substrate specificity [39]. Nevertheless, analysis of yeast *i*-AAA protease indicated the presence of a specific motif of amino acids (F-*h*-*h*-F, where *h* stands for any hydrophobic residue) that in unfolded state may serve as a degron sequence required for the *i*-AAA-dependent degradation [37,40]. Correspondingly, sequences that are recognized by *m*-AAA protease also seem to contain some characteristic hydrophobic residues [41].

Regulation of proteolysis can be achieved through the action of protease-specific binding partners, including adaptor proteins that are able to increase the substrate recognition abilities of their related protease. While for both, *i*-AAA and *m*-AAA proteases, interacting proteins have been identified, their significance for the proteolysis is still not entirely understood. In all eukaryotes *m*-AAA protease forms supramolecular structures with prohibitin complexes [35,42,43]. In yeast and mammals, two homologous prohibitin subunits, Phb1/PHB1, and Phb2/PHB2 form a large heterodimeric ring-shaped complex in IM that subsequently associate with *m*-AAA. Deletion of either of the prohibitin subunits in yeast results in increased turnover of unassembled Cox3 by *m*-AAA [42]. Thus, PHB complexes negatively regulate the activity of *m*-AAA protease. In addition, mammalian *m*-AAA protease interacts with the matrix protein MAIP1 [44]. Interestingly, MAIP1 protects precursor of the subunit of mitochondrial Ca^2+^ uniporter (MCU), EMRE, from the *i*-AAA mediated degradation [44]. *i*-AAA protease is also a part of supramolecular assemblies in IM. Yeast *i*-AAA protease interacts with Mgr1 and Mgr3 that seem to facilitate protease function [45,46]. Mgr1 and Mgr3 behave analogously to adaptor proteins enhancing association between substrates and the protease [23,45,46]. Thus far, no homologs of Mgr1 and Mgr3 proteins were identified in higher eukaryotes. Instead, mammalian *i*-AAA protease forms supramolecular complex, named SPY, together with a membrane scaffold stomatin-like protein SLP2, and the rhomboid protease PARL. SLP2 in SPY complex regulates the cleavage of PINK kinase by PARL [47]. Conversely, the functional relationships between *i*-AAA protease and other constituents of SPY complex still remain to be clarified. Interestingly, amongst proteins interacting with plant *i*-AAA protease, counterparts of mammalian SLP2, Slp1, and Slp2, were also identified [48]. 

The *m*-AAA and *i*-AAA proteases differ enormously in their topologies. The number of encoded transmembrane segments (TMs) determines the opposite orientations of AAA proteases across the IM (Figure 1B). While single transmembrane region imposes IMS location of the catalytic domains of *i*-AAA protease, the presence of additional membrane spanning region in *m*-AAA subunits exposes the catalytic domains to the matrix [23]. Thus, the spectrum of AAA proteases substrates not only includes IM constituents, but also matrix or IMS localized proteins, for *m*-AAA and *i*-AAA, respectively. Recent findings in yeast indicate that also OM-anchored proteins can be degraded by *i*-AAA, extending the range of AAA proteases substrates to components of virtually all mitochondrial sub-compartments [49]. AAA proteases completely remove recognized proteins from mitochondria by breaking them into small peptide fragments [39]. Albeit, structural constrains may limit proteolysis and lead to proteolytic processing of substrates by these ATP-dependent proteolytic machines [50]. In addition to degradation or processing of proteins, AAA proteases are also able to perform protein dislocation from the membrane without proteolysis [23]. Consequently, AAA proteases, by astonishingly versatile activities, influence multiple life-sustaining processes within mitochondria.

## 3. AAA Proteases Maintain Functional and Healthy Mitochondria

### 3.1. Role of AAA Proteases in Protein Mitochondrial Quality Control (PMQC)

Due to intrinsic exposure of proteome to challenging conditions, mitochondria are equipped with mitochondrial quality control system (MQC) that counteracts the potential organellar injuries. Accordingly, MQC is based on multiple independent mechanisms operating at both molecular and organellar/cellular levels [13,51]. The molecular MQC (or protein MQC (PMQC)) is composed of chaperones and proteases that jointly survey, repair, or remove damaged and superfluous mitochondrial proteins within mitochondrial sub-compartments [52]. Cytosolic machineries, including the ubiquitin-proteasome system (UPS), parallelly contribute to the maintenance of proteostasis in IMS and OM [53,54]. Remarkably, PMQC may also function beyond controlling mitochondrial proteome supporting cytosolic protein homeostasis as well [55].

The key house-keeping role of PMQC involves surveillance of the biggest mitochondrial threat, the ROS-generating OXPHOS system. The rapid removal of aberrant OXPHOS constituents impedes accumulation of aggregation-prone polypeptides that have negative impact on mitochondrial function leading to the uncontrolled formation of deleterious ROS [13,30,32,56,57,58,59,60,61]. AAA proteases are central for the implementation of this task. Across diverse species, both *m*-AAA and *i*-AAA proteases remove aberrant components of respiratory chain complexes or F_1_-F_0_ ATP synthase at both sides of mitochondrial inner membrane [13,30,32,56,57,58,59,60,61]. Oxidatively damaged mitochondrial proteins arising as a result of imbalanced OXPHOS functioning can also be degraded by AAA proteases [48,60,62,63]. AAA proteases persistently monitor IM against superfluous subunits, whose accumulation may interfere with the assembly and activity of the complex and subsequently compromise mitochondrial function. In addition, it was reported that *m*-AAA protease independent of its proteolytic function is able to use the chaperone-like activity to facilitate assembly of the membrane-associated ATP synthase subunits [32]. AAA proteases contribute to optimal formation and maintenance of OXPHOS, but also other vital IM complexes, including mammalian mitochondrial calcium uniporter (MCU) and mitochondrial cristae organizing system (MICOS) [10,23,25,44,64]. Correspondingly, genetic loss of any of AAA proteases is associated with severe pleiotropic phenotypes, including defects in respiratory function and in mitochondrial morphology as well as oxidative stress [10,13,23,24,25,65,66]. Depending on species/tissue context, the absence of the AAA proteases-dependent surveillance may have diverse consequences, including cell death in the worst-case scenario. For instance, *m*-AAA malfunction in mammals is linked to substantial neuronal loss [62,67,68,69,70,71,72]. Recent studies indicated that neuronal death triggered by *m*-AAA protease deficiency is mainly a consequence of the deregulation of mitochondrial Ca^2+^ homeostasis [44,67,68]. In mammalian neurons, Ca^2+^ ions are transported into mitochondrial matrix through IM-bound MCU channels [73]. The activity of MCU must be tightly modulated, otherwise mitochondrial Ca^2+^ overload and cell death may occur. Mammalian MCU is formed by channel-forming MCU subunits, small protein EMRE and a gatekeeper subunits MICU. The gating of MCU complex requires association between EMRE and MICU subunits [74,75,76]. However, unassembled EMRE subunits hijack MICU regulatory proteins from MCU resulting in the formation of constitutively open MCU complexes and subsequent Ca^2+^-induced cell death. Recent studies revealed that removal of unassembled EMRE subunits by *m*-AAA protease is necessary to prevent these catastrophic consequences [44,67].

The house-keeping function of AAA proteases is not only restricted to IM proteome. The *i*-AAA protease constitutes a key element of PMQC of IMS. IMS is a relatively small mitochondrial sub-compartment containing from 50 to 130 proteins in yeast and mammals, respectively [77,78]. Majority of IMS proteins contain conserved cysteine motifs that are necessary for their proper folding including formation of disulfide bonds [79]. Oxidative stress can result in failure in this process and consequently lead to accumulation of covalently-linked aggregates. Studies in yeast demonstrated that *i*-AAA protease prevents aggregation of variety of IMS-localized proteins [80]. Strikingly, Yme1 achieves this not only by the removal of misfolded proteins, but also by the chaperone-like activity of its AAA domain [81,82]. Amongst IMS proteins proven to be under tight Yme1-dependent surveillance are components of mitochondrial protein import machinery, namely small TIM proteins (Tim9 and Tim10). In vitro studies using solubilized Yme1 protease showed a correlation between disulfide bond disruption and increased rate of *i*-AAA-dependent degradation for both small TIM proteins [40].

### 3.2. Regulated Proteolysis: Fine-Tuning of Mitochondrial Pathways

The function of AAA proteases is not only restricted to PMQC. More and more studies indicate that these ATP-dependent machineries similarly to their bacterial ancestor, FtsH, are also capable of performing highly specific and regulated proteolysis of natively folded regulatory proteins [10,83]. The careful management of the life span of regulatory proteins warranties mitochondrial plasticity that is essential for rapid organellar adaptation to changing environmental conditions [10,25]. Turnover of regulatory proteins mediated by AAA proteases modulate key processes, including mitochondrial biogenesis or stress responses [10,25].

Mitochondrial biogenesis not only requires synchronized supply of proteins, but also phospholipids, which are fundamental building blocks of their membranes. Mitochondrial membranes comprise phospholipids that either must be imported (phosphatidylcholine, phosphatidylinositol, phosphatidylserine, phosphatic acid) or synthetized inside the organelle from the precursor, phosphatic acid, (phosphatidylglycerol, phosphatidylethanolamine, cardiolipin) [84]. Cardiolipin (CL) is a unique phospholipid found almost completely in the IM, where it has a significant impact on many vital processes, including mitochondrial dynamics, respiration, protein import, and apoptosis [84,85]. The abnormalities in phospholipid levels are associated with mitochondrial malfunction and may lead to development of severe diseases [84,85,86]. Therefore, mitochondria are equipped with diverse mechanisms destined to fine-tune phospholipids amounts in their membranes [87].

The yeast and mammalian *i*-AAA proteases control mitochondrial membrane composition by defining the half-lives of IMS-located lipid carrier proteins belonging to MSF’/PRELI family, they degrade natively-folded Ups1/Ups2 or PRELID1, respectively [88,89]. The *i*-AAA protease-dependent removal of Ups1/Ups2/PRELID1 negatively regulates cardiolipin levels in mitochondria [88,89]. In yeast, both Ups1 and Ups2 form complexes with Mdm35 that are required for the transfer of phosphatic acid, precursor of phosphatidylethanolamine and cardiolipin, from OM to IM [90,91]. It was postulated that high concentration of CL in the IM stimulates the binding of Ups1-Mdm35 to IM promoting Yme1-dependent degradation. Whereas, low levels of CL in IM results in the reduced association of Ups1-Mdm35 complex to IM and its subsequent shuttling to OM [90,91]. Interestingly, *Arabidopsis thaliana* devoid of functional FTSH4 was reported to have altered levels of cardiolipin suggesting that plant *i*-AAA protease by yet unknown mechanisms is also taking part in the maintenance of phospholipid homeostasis [92].

Notably, AAA proteases through regulated proteolysis also modulate other biogenic pathways. Adaptation of mitochondrial protein import pathways emerged as a significant way of preserving mitochondrial proteostasis and function during stress [93,94,95,96,97]. Mammalian *i*-AAA protease efficiently attenuates mitochondrial protein import during stress by the removal of Tim17A subunit of TIM23 translocase [94]. Downregulation of TIM23-dependent import pathway, utilized by two-third of mitochondrial precursors, leads to the substantial reduction in the burden of newly-synthetized unfolded polypeptides entering the organelle [98]. This can promote mitochondrial proteostasis by increasing the capacity of PMQC that is available inside the organelle. In addition, reducing of TIM23-dependent import promotes the induction of mitochondrial Unfolded Protein Response-associated genes [94]. In plant mitochondria, the counterpart of mammalian Tim17A, Tim17-2, was identified as a substrate of *i*-AAA protease, FTSH4 [99]. Tim17-2 is distinguished from other plant mitochondrial protein import components by relatively fast turnover rate [100]. Similarly, to mammalian *i*-AAA protease FTSH4 negatively regulates TIM23-complex dependent import by controlling Tim17-2 levels [99]. However, the factors that trigger this *i*-AAA-dependent proteolysis in plant mitochondria still remain to be elucidated.

### 3.3. AAA Proteases: Much More beyond the Machinesof Protein Destruction

Maintenance of mitochondrial function and homeostasis depends enormously on modulation of activities of proteins by their incomplete proteolysis. First, the vast majority of mitochondrial proteins are imported into the organelle as pre-proteins and their activation requires removal of targeting sequences [98]. Second, proteolytic processing is central to activate or inhibit components of pathways regulating mitochondrial dynamics, mitophagy or programmed cell death [10].

As previously stated, AAA proteases are capable of, in addition to substrate removal, highly specific proteolytic processing. The best-known example of this mode of action is the *m*-AAA protease-dependent maturation of nuclear-encoded subunit of mitochondrial ribosome, MrpL32 [50,101,102]. This mechanism is highly conserved in diverse eukaryotes, including yeast, mammals and plants [50,101,102,103,104]. In yeast, 71 amino acids from the unstructured N-terminus of newly imported MrpL32, containing mitochondrial-targeting sequence, are removed by *m*-AAA protease in mitochondrial matrix [50]. This step is essential for the assembly of functional mitoribosome and activation of mitochondrial protein translation. In yeast, proteolytic processing of MrpL32 precursor appear to be the most central function of *m*-AAA protease in mitochondria. The success of this highly specific *m*-AAA-dependent processing is based on the presence of a conserved metal-binding cysteine motif in the C-terminal part of MrpL32 that provides structural constraints for further proteolysis. This leads to *m*-AAA protease stall that triggers subsequent release of mature MrpL32 subunit [50]. Importantly, mitoribosome assembly is also impaired in vivo in the brain of mice lacking *m*-AAA subunit, AFG3L2 [103]. In addition, mammalian *m*-AAA protease subunits undergo autoproteolytic cleavage after the presequence removal by the mitochondrial processing peptidase (MPP) [105].

*i*-AAA protease mediates partial substrate processing that regulates vital mitochondrial processes as well. In yeast, *i*-AAA protease controls vacuolar-dependent removal of damaged mitochondria in processes known as mitophagy. The cleavage by Yme1 of C-terminal IMS-located domain of OM-anchored mitophagy receptor protein, Atg32, initiates subsequent organellar degradation [106]. On the other hand, mammalian *i*-AAA protease by mediating incomplete proteolysis of IM-localized dynamin-like GTPase, OPA1, also directly controls central mitochondrial quality and homeostasis pathways [107,108]. Mitochondria are highly dynamic organelles. Continuous fission and fusion events preserve the optimal functioning of mitochondria [109,110]. Whereas fission enable segregation and removal of damaged mitochondrial constituents through mitophagy, fusion facilitates exchange of material between the healthy organelles [11,107,108,109,110]. The balance between mitochondrial fusion and fission is regulated by the relative abundance of unprocessed OPA1 (L-OPA1) and its shorter forms (S-OPA1) [107,108,109,110]. The sequential proteolytic processing of OPA1 by OMA1 and YME1L initiates formation of OPA1 short forms that can subsequently trigger mitochondrial fission [107,108,109,110]. Interestingly, loss of YME1L in cultured fibroblasts was demonstrated to trigger OMA1-dependent mitochondrial fragmentation as well [107,108]. Furthermore, conditional mouse model characterized by cardiac-specific deletion of YME1L displays cardiac mitochondrial fragmentation that is linked to cardiomyopathy and heart failure [66]. The balance between fusion and fission is probable also disturbed in plants lacking FTSH4 due to the presence of giant mitochondria, however, mechanism linking abnormal mitochondrial morphology with the loss of plant *i*-AAA is currently poorly understood [92].

**Figure 2:**
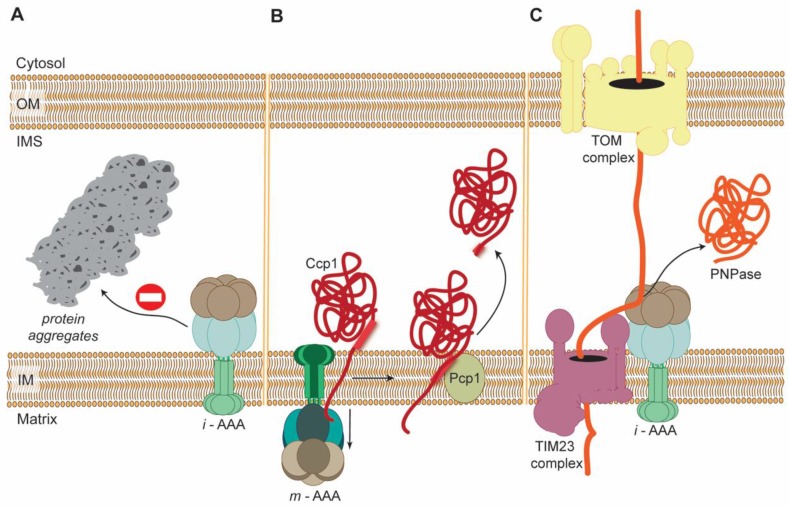
Examples of actions of yeast AAA proteases that are independent of their proteolytic activity. (**A**) *i*-AAA protease prevents aggregation of misfolded intermembrane space proteins through chaperone-like activity. (**B**) *m*-AAA dislocates precursor of Ccp1 from the mitochondrial inner membrane (IM) enabling its cleavage by the rhomboid protease Pcp1. The membrane dislocation step is dependent on ATPase activity of *m*-AAA protease. (**C**) *i*-AAA protease independently of its proteolytic activity mediates import of heterologously expressed mammalian PNPase precursor into mitochondrial intermembrane space (IMS). The transfer of PNPase precursor from the cytosol into organelle is dependent on the tight cooperation between *i*-AAA protease, the main translocase of the outer membrane (OM), TOM complex, and the translocase of the inner membrane, TIM23 complex.

Finally, AAA proteases can, independently of proteolytic activities, execute membrane dislocation of particular substrates (Figure 2). For instance, yeast *m*-AAA protease was demonstrated to be required for the maturation of IMS-localized ROS scavenging enzyme, cytochrome c peroxidase (Ccp1) [111]. Ccp1 precursor during its import into mitochondria is anchored in IM. Release of mature Ccp1 enzyme to IMS requires cleavage by IM rhomboid protease Pcp1. Remarkably, Pcp1-dependent proteolytic processing of Ccp1 precursor involves its dislocation from the IM. This energy-demanding step of Ccp1 maturation is driven by *m*-AAA protease [111]. Analogously, *i*-AAA protease is also competent to facilitate, independent of its proteolytic activity, the import of heterologously expressed mammalian polynucleotide phosphorylase (PNPase) into the IMS of yeast mitochondria [112] (Figure 2). As mentioned earlier, yeast *i*-AAA protease displays, in addition to its proteolytic function, also chaperone-like activity towards many IMS-localized aggregation-prone proteins [80] (Figure 2).

## 4. AAA Proteases in the Pathogenesis of Human Diseases

Versatile activities that are executed by AAA proteases ensure optimal mitochondrial functioning. Respectively, disturbances in the performance of these ATP-dependent enzymes are associated with decline in mitochondrial health leading to development of many pathological conditions [10,23,67]. Studies using mouse models indicated that depletion in activities of AAA proteases might result in malfunction of critical cellular processes, including decline in activity of OXPHOS complexes, impairment in mitochondrial translation, disturbances in mitochondrial morphology, calcium deregulation as well as dysfunction of mitochondrial anterograde transport [62,66,67,68,69,70,71,72]. Mitochondrial abnormalities predominantly influence organs and tissues with the high energetic requirements contributing to onset of cardiovascular, neurodegenerative, or complex metabolic diseases such as type 2 diabetes mellitus [10,23,67].

Many mutations were identified in genes encoding subunits of AAA proteases that are associated with neuronal loss and diverse hereditary neurodegeneration diseases. For instance, clinical studies revealed occurrence of homozygous mutation in *YME1L* that causes defects in maturation of human *i*-AAA protease resulting in infantile-onset mitochondriopathy with optic atrophy [113]. Similarly, various amino acid substitutions in *m*-AAA protease subunits, AFG3L2 or paraplegin (SPG7), are associated with development of severe neurological diseases [114,115,116,117,118,119,120,121,122,123]. The absence of SPG7 subunit impairs only heteromeric *m*-AAA protease, while loss of AFG3L2 has more dramatic consequences as it disturbs both homo and heteromeric forms of *m*-AAA protease [67]. At least 17 amino acid substitutions in AFG3L2 have been linked to autosomal dominantly inherited juvenile-onset spinocerebellar ataxia type 28 (SCA28) [115,117,118,119,120,121]. SCA28 is associated with Purkinje cell loss and is characterized by slurred speech, lack of limb coordination and eye movement abnormalities [123]. On the other hand, mutations in *SPG7* are associated with autosomal recessive hereditary spastic paraplegia (HSP). This mitochondriopathy is characterized by spasticity and weakness of lower limbs. Amyotrophy, cortical and cerebellar atrophy as well as mental retardation can also occur in HSP [122]. Moreover, mutations in *SPG7* are also associated with defects in mtDNA maintenance triggering chronic ophthalmoplegia [124,125]. Clinical features of both SCA28 and HSP7 occur in severe, early onset spastic ataxia 5 (SPAX5) that can be initiated by recessive mutations in *AFG3L2* causing hampered interactions between AFG3L2 and SPG7 subunits [116]. Furthermore, single nucleotide polymorphisms in *SPG7* have been recently linked to the development of multi-system diseases such as type 2 diabetes mellitus and coronary artery disease [126].

## 5. Final Remarks

**Figure 3:**
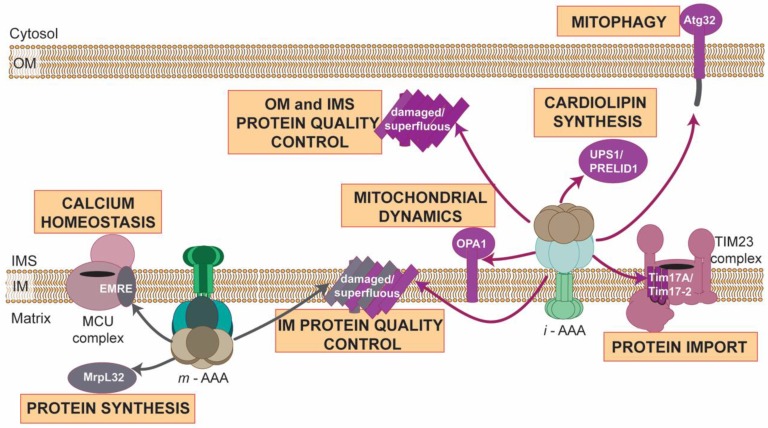
Multifaced activities of AAA proteases at mitochondria. Illustration summarizes spectrum of known proteolytic substrates of either yeast, mammalian or plant *i*-AAA (violet arrows) or *m*-AAA proteases (grey arrows), whose either processing or removal by AAA proteases modulate diverse mitochondrial functions. OM, outer membrane; IMS, intermembrane space; IM, inner membrane.

Our understanding of roles of AAA proteases in mitochondria has transformed in a recent decade. AAA proteases are no longer viewed as a simple machines of protein destruction that are exclusively required for the removal of damaged or unfolded polypeptides from mitochondria. Instead, AAA proteases can be considered guardians of mitochondrial function and homeostasis, which, in addition to proteome quality survey, specifically modulate fundamental mitochondrial processes by controlling levels of regulatory proteins and activating or inhibiting critical components of essential mitochondrial pathways (Figure 3).

Despite of enormous progress, there are still many questions that remain to be answered to fully appreciate significance of AAA proteases for maintenance of mitochondrial function and homeostasis. Pleiotropic phenotypes observed upon loss of either of AAA proteases suggest that only a tiny portion of the actual repertoire of mitochondrial constituents that is directly controlled by these ATP-dependent enzymes is known. Unveiling a complete catalogue of physiological substrates of AAA proteases both under basal and homeostasis-disturbing conditions is one of the main challenges for future studies. Likewise, understanding of the molecular basis behind the astonishing plasticity displayed by these enzymes awaits further survey. This certainly requires identification of regulatory mechanisms that modulate AAA proteases activity and specificity. Ultimately, this knowledge can shed light on pathogenesis of many mitochondria-related diseases and essentially contribute to development of novel therapeutic strategies.
